# An Interactive Internet-Based Plate for Assessing Lunchtime Food Intake: A Validation Study on Male Employees

**DOI:** 10.2196/jmir.2217

**Published:** 2013-01-18

**Authors:** Madeleine Svensson, Rino Bellocco, Linda Bakkman, Ylva Trolle Lagerros

**Affiliations:** ^1^Karolinska InstitutetDepartment of MedicineUnit of Clinical EpidemiologyStockholmSweden; ^2^School of Health and Social SciencesHalmstad UniversityHalmstadSweden; ^3^Karolinska InstitutetDepartment of Medical Epidemiology and BiostatisticsStockholmSweden; ^4^University of Milano-BicoccaDepartment of StatisticsMilanItaly

**Keywords:** diet, epidemiology, Internet, methods, nutrition, validation, Web

## Abstract

**Background:**

Misreporting food intake is common because most health screenings rely on self-reports. The more accurate methods (eg, weighing food) are costly, time consuming, and impractical.

**Objectives:**

We developed a new instrument for reporting food intake—an Internet-based interactive virtual food plate. The objective of this study was to validate this instrument’s ability to assess lunch intake.

**Methods:**

Participants were asked to compose an ordinary lunch meal using both a virtual and a real lunch plate (with real food on a real plate). The participants ate their real lunch meals on-site. Before and after pictures of the composed lunch meals were taken. Both meals included identical food items. Participants were randomized to start with either instrument. The 2 instruments were compared using correlation and concordance measures (total energy intake, nutritional components, quantity of food, and participant characteristics).

**Results:**

A total of 55 men (median age: 45 years, median body mass index [BMI]: 25.8 kg/m^2^) participated. We found an overall overestimation of reported median energy intake using the computer plate (3044 kJ, interquartile range [IQR] 1202 kJ) compared with the real lunch plate (2734 kJ, IQR 1051 kJ, *P*<.001). Spearman rank correlations and concordance correlations for energy intake and nutritional components ranged between 0.58 to 0.79 and 0.65 to 0.81, respectively.

**Conclusion:**

Although it slightly overestimated, our computer plate provides promising results in assessing lunch intake.

## Introduction

Measuring food intake is a challenge. Most assessment tools rely on an individual’s ability to accurately recall and report foods consumed, usually according to a fixed format of an instrument [1]. Examples of traditional methods to examine food intake include food frequency questionnaires (FFQ), 24-hour recalls, and food recording and weighing [1-3], as well as the duplicate-portion technique [4]. Weighing individuals’ food plates with individually composed meals before and after eating is the most precise method, but it can be a rather costly, time-consuming, and impractical approach. Hence, self-reported food intake is typically used in health screenings.

One of the challenges of self-reported food intake is the high rate of misreporting [5,6]. Overweight or obese women and individuals of low socioeconomic status [7] tend to underreport food consumption. Food items that are sweet, fatty, and considered unhealthy are more likely to be underreported. In contrast, food with high protein content or vegetables and fruits are frequently exaggerated [3,7-12]. Although some validation studies present accurate measurements of food intake [1], respondents’ may still struggle with reporting food intake because of extensive questionnaires that are difficult to fill out [8].

A recent study conducted by Illner et al [13] reports a similar degree of misreporting of food intake irrespective of method of delivery. More specifically, the participants’ food intake reporting was identical using paper-based frequency assessments and technology-based assessments (ie, Internet-based). Yet, the benefits gained from using technology in food assessments may speak for an increased interest and usage in nutritional research [13,14] compared with conventional methods. The Internet promotes time- and cost-effective research and facilitates administration of research material, as well as collection and storage of data [14]. In addition, it allows for interactivity that, in turn, produces opportunities for the development of pedagogical advancements [15].

Pictures of foods and meal compositions have been used to facilitate reporting of food intake in prior nutritional research [16,17]. For instance, Turconi et al [16] asked their study participants to estimate food intake by looking at pictures of prepared meals of different portion sizes (small, medium, and large) put together in food atlases. The estimated meals were then compared with the participants’ intake of actual meals, and indicated promising results on the participants’ overall comprehension of food intake. Elinder et al [18] also reported valid results from allowing individuals with intellectual disabilities to photograph meals before and after intake. Hence, the use of pictures seems as an appropriate strategy in food intake assessments.

To incorporate the advantages of technology and visuals in nutritional research, we developed an Internet-based virtual food plate to measure lunch intake using the computer. Our computerized food plate allows for interactive composition of a single lunch meal, in which the user can add or subtract pictures of food items onto a virtual plate. To our knowledge, this format of food intake assessments has not been described previously. Consequently, the present study aimed to validate our new instrument against the golden standard—the participants’ real lunch meal composition using real food items and utensils.

## Methods

### Participants

Between February and April 2010, 56 male employees (age 18-65 years) at the Swedish Transport Administration, Stockholm, Sweden, were asked to participate in the study. The predetermined food items of our instrument did not include vegetarian protein sources; therefore, one potential study participant who reported being a vegetarian was excluded from participating. Hence, a total of 55 employees participated in the present study. Most of the participants were employed as engineers (ie, work in an office).

### Study Design

This validation study included two parts, using identical food items. The participants were asked to compose a lunch meal that represented their usual intake by means of (1) an interactive Internet-based food plate by adding suggested food items onto a virtual plate on the computer (hereafter referred to as “computer plate”), and (2) an ordinary lunch plate by adding real food items onto a real plate during a lunch setting (hereafter referred to as “real lunch plate”).

**Figure 1:**
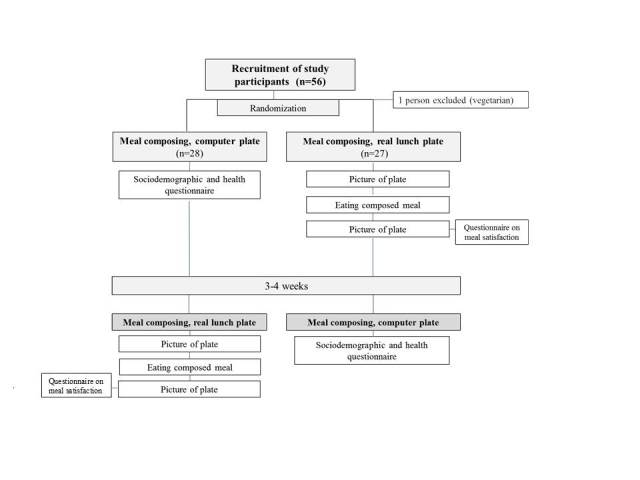
Flowchart of the validation process.

The participants were recruited by the researchers in the company’s main lobby during lunch hours (10 am - 1 pm). Upon recruitment, 28 participants were asked to start with the computer plate and 28 participants were asked to start with the real lunch plate. They were instructed to complete the remaining part (the computer plate or the real lunch plate) after 3 to 4 weeks. See Figure 1 for a flowchart of the study design. All participants signed an informed consent form prior to study start. The study was approved by the Karolinska Institutet’s Ethical Committee in Stockholm, Sweden.

### The Computer Plate

**Figure 2:**
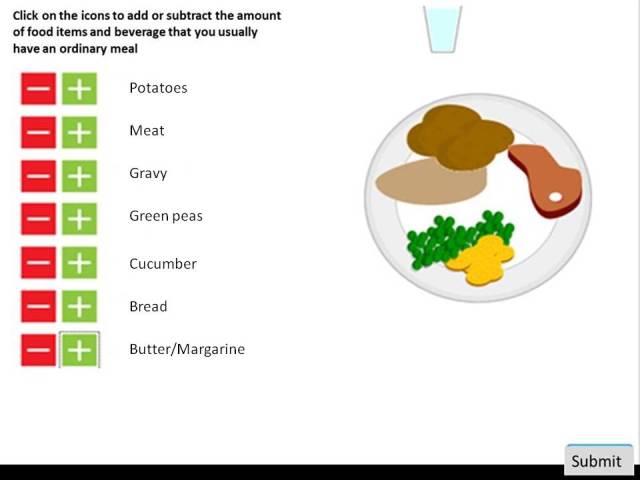
Example of the computer plate and listed food items.

We developed a computer plate, an interactive instrument designed as a virtual food plate, available on a website. The virtual plate was placed in the center of the Web page, with a list of food items to the left. By clicking on the “+” and “–” buttons with the computer mouse, computer-generated pictures of food items were added to or subtracted from the virtual plate. It was possible to increase the quantity (or vice versa) of a food item by clicking several times. The participants had 7 food items to choose from when composing their meal on the computer, including boiled potatoes, meat (pork chops), gravy, green peas, slices of cucumber, slices of bread, and butter. Five different beverages were offered, including light beer (<3.5% alcohol), strong beer (≥ 3.5% alcohol), juice, milk (1.5% fat), and water. The food items were chosen because they are commonly represented in a Swedish lunch meal. See Figure 2 for an illustration of a composed lunch meal using the computer plate. (All beverages are not visible in Figure 2, but they appeared in the upper right corner of the website upon completion of the lunch meal assessment).

### Study Part 1: The Computer Plate

The participants arrived at the main lobby and were instructed on how to complete the computer plate on a laptop provided by the researchers. The participants were informed that they could only build one plate representing a usual lunch meal, meaning that if they usually refilled their lunch plates with food, this refill had to be considered when composing the virtual meal. When the meal was composed, we saved the screen picture of the lunch meal in a Word document.

Next, the participants completed a questionnaire on sociodemographics, body mass index (BMI), physical activity level (PAL) [19], smoking habits, food allergies, and intake of breakfast or snacks prior to composing their computerized lunch meal.

### Study Part 2: The Real Lunch Plate

The participants arrived to their workplace lunch area at lunchtime. Again, the participants were informed that they could only build one plate of a usual lunch meal (if they typically refilled their lunch plates, this amount of food had to be considered when composing the real lunch meal). The participants ate their lunch meal in the lunch area. Photos of the composed meals were taken prior and subsequent to eating.

After the participants composed their lunches, they were asked to fill out a questionnaire (described previously). In addition, they were asked to rate their level of fullness on a scale of 1 to 10 [20] to examine whether their reported lunch intake represented an appropriate meal intake.

The meals were not weighed because the purpose of our study was to examine whether the participants were able to visualize an ordinary lunch meal by using the pictures of food items provided in our computer plate instrument. We focused on the quantity of food items and overall nutritional content rather than the weight of food.

### Statistical Analyses

We excluded 4 participants because of incomplete data. Descriptive statistics, such as median and interquartile range (IQR), were computed to summarize the participants’ characteristics (eg, age, BMI, PAL, and meal satisfaction). Further, we computed summary statistics of the participants’ composed meals with respect to total energy intake in kilojoules (kJ), as well as quantity of food items and nutritional components, including total energy intake (EI) and energy percentage (E%) of carbohydrates, proteins, and fats of the 2 meals. The before and after pictures of the composed meals were used to calculate the participants’ quantities of food items included in the lunch meal.

We focused our analyses on total EI from food items on the plate excluding energy from beverages and bread/butter. The reason for this focus was because of the large energy differences among the various beverages to choose from (ie, the low/zero energy from water versus the high energy from beer). Wilcoxon signed rank tests and Wilcoxon rank sum tests [21] were used to test the difference between medians of the computer plate and real lunch plate measurements, and to assess if such differences were dependent on variables and/or on specific participant characteristics.

Participants were categorized into two groups based on the order of completing the 2 meal assessments: (1) computer plate-real lunch plate or (2) real lunch plate-computer plate. The participants were also categorized according to their BMI as normal weight (< 25 kg/m^2^) or overweight (≥ 25 kg/m^2^). Age was categorized as < 45 years or ≥ 45 years to study any differences in these characteristics with respect to meal composition. The cutoff age of 45 years was chosen because the median age was 45 years.

We used the Spearman rank correlation (ρ) to study the linear association among the reported EI, nutritional components, and food items using the computer plate and real lunch plate. The Spearman rank correlation is a nonparametric test that ranks 2 sets of outcomes distinctly and calculates a coefficient of rank correlation [22]. To further study the associations, we used the concordance correlation coefficient (ρ_c_), an intraclass correlation that duplicates readings as replicates (random) rather than two distinct readings. It “evaluates the agreement between the two readings by measuring the variation from a 45° line through the origin (degree of concordance)” [23]. In addition, Bland-Altman plots were used to assess the differences between means of EI (kJ) estimated from the computer plate and the real lunch plate, plotted against the mean energy intake from the 2 methods [24]. To interpret the agreement, we considered participants with reported EI within the interval of ±10% from the mean (3014 kJ) of the real lunch plate as acceptable values of our new instrument.

Correlations and 95% confidence intervals (CI) were computed for all participants and stratified by groups (defined previously). All statistics were computed using the real lunch plate as the golden standard (reference). A significance level of .05 was used. Stata version 12 (Statacorp LP, College Station, TX, USA) was used for all statistical calculations and analyses.

## Results

A total of 51 employees participated in the study. The participants had a median age of 45 years (IQR 21 years), a median BMI of 25.8 kg/m^2^ (IQR 4.18 kg/m^2^), and a median physical activity level of 1.65 PAL (IQR 0.1 PAL) (Table 1).

**Table 1:** Descriptive statistics of the study participants (N=51).

Description	Participants
Sex (males), n (%)	51 (100)
Age (years), median (IQR)	45 (21)
Body mass index (kg/m^2^), median (IQR)	25.8 (4.18)
Physical activity level, median (IQR)	1.65 (0.1)
Smoker, n (%)	2 (3.7)
Had breakfast before study participation, n (%)	48 (87)

The participants’ reported total EI was somewhat higher on the computer plate compared to the real lunch meal. Overall, the median reported EIs for the computer plate and real lunch plate were 3044 kJ (IQR 1202 kJ) and 2734 kJ (IQR 1051 kJ, *P*<.001), respectively (Table 2). Although not significantly different, we noted that the participants with a BMI ≥ 25 kg/m^2^ (Table 3) and those aged ≥ 45 years (data not shown) reported lower overestimations of EI (+147 kJ and +193 kJ, respectively) using the computer plate, compared to their counterparts (BMI < 25 kg/m^2^: +595 kJ, *P*=.90; age < 45 years: +649 kJ, *P*=.33). Also, the EI measured from the computer plate was 172 kJ higher for participants starting with the real lunch plate compared to those starting with the computer plate (+729 kJ), although not statistically significant (*P*=.75).

**Table 2:** Study participants’ composed meals using the two meal instruments (N=51).

Composed meal		Median (IQR)	
		Computer plate	Lunch plate
**Reported intake** ^**a**^			
	Total energy, kJ	3044 (1202)	2734 (1051)
	Total energy including drinks, kJ	3341 (1348)	2989 (1277)
	Total food, g	855 (189)	779 (248)
	Total carbohydrates, E%	128 (57)	134 (67)
	Total carbohydrates, g	59 (24)	55 (18)
	Total proteins, E%	121 (29)	121 (29)
	Total proteins, g	43 (32)	41 (31)
	Total fat, E%	167 (29)	163 (38)
	Total fat, g	30 (19)	26 (17)
**Food items (number of)**			
	Total potatoes	3 (1)	2 (1)
	Total meat, pork chops	1 (1)	1 (1)
	Total green peas, tbsp	2 (2)	3 (1)
	Total gravy, tbsp	2 (1)	1.3 (1)
	Bread, slices	1 (1)	1 (0)
	Cucumber, slices of 5	1 (1)	0.83 (0.5)

^a^ If not noted, reported intake is excluding intake from beverages; E%: energy percentage (in kilojoules).

**Table 3:** Study participants’ composed meals using the two meal instruments, by body mass index (BMI) (N=51).

Composed meal		BMI < 25, median (IQR)		BMI ≥ 25, median (IQR)	
		Computer plate	Lunch plate	Computer plate	Lunch plate
**Reported intake** ^**a**^					
	Total energy, kJ	3320 (1361)	2726 (1101)	2881 (1080)	2734 (1017)
	Total energy including drinks, kJ	3513 (1160)	3061 (1436)	3006 (1022)	2989 (1273)
	Total meal, g	880 (175)	842 (235)	834 (172)	758 (243)
	Total carbohydrates, E%	134 (59)	142 (42)	130 (46)	121 (67)
	Total carbohydrates, g	61 (21)	58 (15)	57 (31)	47 (22)
	Total proteins, E%	121 (33)	117 (21)	121 (25)	126 (29)
	Total proteins, g	58 (33)	41 (32)	42 (32)	41 (32)
	Total fat, E%	167 (33)	151 (29)	167 (25)	167 (38)
	Total fat, g	35 (26)	26 (22)	30 (19)	26 (16)
**Food items (number of)**					
	Total potatoes	3 (1)	3 (1)	3 (1)	2 (1.5)
	Total meat, pork chops	1.5 (1)	1 (1)	1 (1)	1 (1)
	Total green peas, tbsp	2 (2)	3 (1.5)	3 (2)	3 (1)
	Total gravy, tbsp	1.5 (1)	1.6 (1)	2 (1)	1.3 (1.3)
	Bread, slices	1 (1)	1 (0)	1 (1)	1 (1)
	Cucumber, slices of 5	1 (2)	0.8 (0.7)	2 (1)	0.7 (0.7)

^a^ If not noted, reported intake is excluding intake from beverages; E%: energy percentage (in kilojoules).

**Figure 3:**
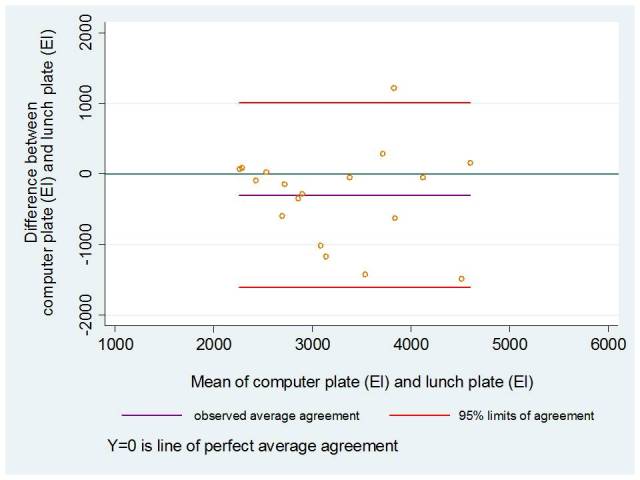
Bland-Altman plot of differences between total energy intake (EI, kJ) of the computer plate and the real lunch plate against the mean of EI (kJ) for each participant with a BMI < 25 kg/m2 (n=18).

**Figure 4:**
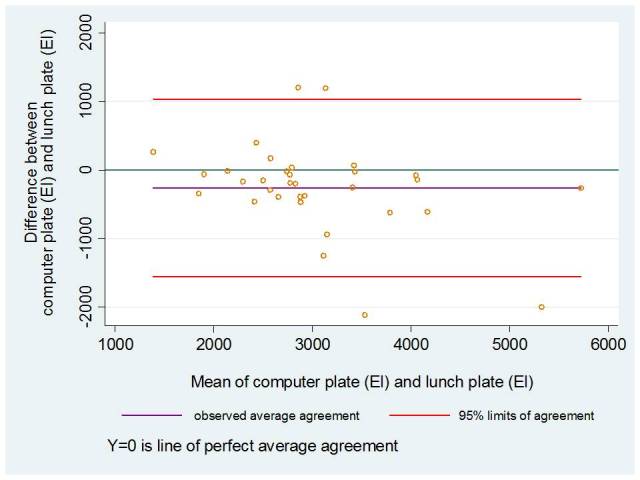
Bland-Altman plot of the differences between total energy intake (EI, kJ) of the computer plate and the real lunch plate against the mean of EI (kJ) for each participant with a BMI ≥25 kg/m2 (n=33).

The quantities of the participants’ chosen food items were similar between the 2 instruments. Only green peas differed, with an underestimation of 1 tablespoon when using the computer plate compared to the real lunch plate (Table 2). Using the Bland-Altman statistics, we found a tendency of agreement in mean EI within the ±10% kJ interval. More than 60% of the normal weight participants’ EIs were represented in this interval. Among the overweight participants, a stronger pattern of agreement was found, with 78% of the participants’ mean EIs represented in this interval (Figures 3 and 4).

Overall, our Spearman rank correlations and concordance correlations between the instruments were both equal to 0.70 for total EIs (slightly higher when including drinks in the calculations), ρ=0.59 and ρ_c_=0.76 for carbohydrates, ρ=0.70 and ρ_c_=0.81 for proteins, and ρ=0.58 and ρ_c_=0.66 for fat (Table 4). All correlations were significant. Further, correlations for specific food items between the 2 instruments ranged from 0.46 to 0.71 for Spearman rank correlations and 0.47 to 0.72 for concordance correlations, with the lowest correlations for gravy and slices of cucumber (Table 4).

Overall, we noted somewhat higher correlations of reported number of food items for those with a BMI ≥ 25 kg/m^2^ (Table 5).

**Table 4:** Spearman rank correlations (ρ) and concordance correlation coefficients (ρ_c_) between the participants’ composed meals using the two meal instruments (N=51).

Composed meal		ρ^a^	ρ_c_^a^
**Reported intake** ^**b**^			
	Total energy, kJ	0.70	0.70
	Total energy including drinks, kJ	0.79	0.72
	Total meal, g	0.72	0.68
	Total carbohydrates, kJ	0.59	0.76
	Total carbohydrates, g	0.69	0.75
	Total proteins, kJ	0.70	0.81
	Total proteins, g	0.71	0.71
	Total fat, kJ	0.58	0.66
	Total fat, g	0.63	0.65
**Food items (number of)**			
	Potatoes	0.65	0.72
	Meat, pork chops	0.69	0.70
	Green peas, tbsp	0.65	0.47
	Gravy, tbsp	0.48	0.50
	Bread, slices	0.71	0.67
	Cucumber, slices of 5	0.46	0.47

^a^ All are statistically significant (*P*<.05).

^b^ If not noted, reported intake is excluding intake from beverages.

**Table 5:** Spearman rank correlations (ρ) and concordance correlation coefficients (ρ_c_) between participants’ composed meals using the two meal instruments by body mass index (BMI).

Composed meal		BMI < 25 (n=18)		BMI ≥ 25 (n=33)	
		ρ^a^	ρ_c_^a^	ρ^a^	ρ_c_^a^
**Reported intake** ^**b**^					
	Total energy, kJ	0.76	0.63	0.67	0.73
	Total energy including drinks, kJ	0.76	0.64	0.82	0.74
	Total meal, g	0.68	0.70	0.70	0.66
	Total carbohydrates, kJ	0.34	0.44	0.70	0.85
	Total carbohydrates, g	0.49	0.60	0.77	0.78
	Total proteins, kJ	0.60	0.59	0.75	0.86
	Total proteins, g	0.76	0.59	0.69	0.76
	Total fat, kJ	0.42	0.30	0.66	0.79
	Total fat, g	0.57	0.52	0.68	0.71
**Food items (number of)**					
	Potatoes	0.38	0.31	0.75	0.79
	Meat, pork chops	0.59	0.55	0.74	0.77
	Green peas, tbsp	0.70	0.41	0.65	0.53
	Gravy, tbsp	0.49	0.53	0.57	0.50
	Bread, slices	0.62	0.59	0.76	0.71
	Cucumber, slices	0.23	0.41	0.57	0.53

^a^ All are statistically significant (*P*<.05).

^b^ If not noted, reported intake is excluding intake from beverages.

Higher Spearman rank correlations and concordance correlation coefficients for food items were found for those who started with the real lunch plate (0.80 and 0.61), in comparison to those who started with the computer plate, respectively. Regarding the Spearman rank correlations and concordance correlation coefficients in relation to age, we found higher coefficients for potatoes and meat for those participants who were ≥ 45 years, but higher coefficients for peas and gravy for those participants < 45 years (data not shown). Based on the questionnaire about meal satisfaction, the participants reported, on average, a level of 7 (mode 8) on the grading scale (0 = low; 10 = maximum) for fullness after meal intake.

## Discussion

The results of this study support the validity of our novel interactive Internet-based food plate to measure lunch intake. The correlations between the participants’ reported meal intake using the computer plate and the real lunch plate were high. Spearman rank correlations and concordance correlations, representing total reported EIs (excluding beverages and condiments), nutritional components, and food items ranged from 0.46 to 0.72 and 0.47 to 0.81, respectively. The strongest correlations were observed for protein, replicating findings from earlier studies [25].

A meta-analysis of FFQs reported Pearson coefficients ranging from 0.46 to 0.58 for EI. The authors concluded that FFQs including questions about more food items produced better estimates than FFQs with fewer items [26]. Conversely, we found strong correlations by using only a few items in a single meal. Similar correlations for specific nutrients were found in a study using cell phone cameras to document food intake [27]. However, because our instrument provides a new way of measuring food intake, the results from our study are incomparable with most previous studies.

Williamson et al [17] found higher correlations of portion sizes from direct visual estimation of a meal rather than from digital photographs. We report an overestimation using our new method on the computer where pictures of food items serve as an integral part of the meal composing process. Although reporting slightly different results, it should be noted that the participants in the study by Williamson et al estimated portion sizes based on fully prepared meals; we allowed the participants to compose a meal using suggested food items. A greater accuracy of food intake reporting has been found using more pictures of food items than fewer items when composing meals [28]. The use of several pictures of separate food items in our participants’ meal composition is thus supported by previous research.

An important point of discussion is the overestimation, rather than underestimation, of food intake noted in our study. All participants overestimated their EI using the computer plate compared to the real lunch plate. Although not significant, even the overweight participants seemed to report higher EIs, contradicting previous experiences [29,30]. Overweight individuals have been found to underreport food intake, with a greater degree of underreporting with increasing BMI [31]. Yet, our results indicate higher correlations of meal intakes from our instrument for those who were overweight as opposed to our normal weight participants.

The fact that our study included only men, mostly middle-aged, and office workers are major limitations in this study preventing us from expanding our findings to participants who are women, not used to working with the computer, or those characterizing age groups other than in our study sample. Another noteworthy factor of this study is that our study sample seemed healthier (ie, much lower number of smokers) than the general population in Sweden [32], which is a common phenomenon among participants in health research [33]. A healthier lifestyle may have influenced the participants’ ability to report lunch intake, and thus the overall validity and applicability of our study.

Also, we only assessed a lunch meal, including only a sample of food items available in an ordinary complete food intake. Preferably, our computer plate should measure total food intake representing various food items, meal options, and combinations. Future research is strongly recommended to explore the ability of our instrument to assess food intake in its entirety.

Even with the limitations of the current study, there are several strengths of the study design that should be highlighted. First, we validated our new measurement tool by using food items commonly consumed by the Swedish population, facilitating the participants’ relatedness in the reporting process of a usual meal intake. Also, the participants were asked to rate their level of fullness after meal intake, allowing us to verify that their registered meal accounted for an actual intake. Moreover, we conducted the study in the company’s dining hall and used the facility’s own dishware and cutlery. In this way, the participants were familiar with the tools (eg, size of food plate, glasses), colleagues, and study climate, thus minimizing potential reporting bias and perhaps some bias from being observed by the researchers.

Although the participants performed the 2 meal assessments with a 3-week time interval to avoid recall bias [34], we noticed higher correlations of reported EI for the group who started with the real food plate. Therefore, we cannot rule out the presence of recall bias in the present study. Whether this result originates from an enhanced memory of recalling previously reported intake attributable to physically composing a lunch meal (compared to the abstract format of the virtual food plate) is difficult to state. The difference between the two assessments was that the participants ate the real lunch meal. The participants’ experience from eating a meal in the lunch area may, therefore, evoke emotions and experiences contributing to an increased memory of the real lunch meal explaining the higher correlations among this group.

Overall, the results from this study demonstrate promising value in food intake assessments. The concept of our computer plate could be extended to examine estimation of daily food intakes. In addition, it may serve as a pedagogical instrument to teach healthier food habits. In fact, by taking advantage of today’s advancements in technology, our computer plate could also be integrated into smartphone technology [15]. Allowing individuals to report food intake via their smartphones is a way to promote time-effective and more accurate food reporting. Future research should focus on the development of our concept, a virtual computerized food plate, to obtain a complete food intake measurement.

### Conclusion

To incorporate the potential of visual and technological advancements in food intake assessments, we developed an Internet-based interactive virtual food plate to measure lunch intake. The validity of our new instrument was high, thereby producing promising applicability in health research. The concept of our computerized food plate could be further developed to assess a complete food intake.

## Abbreviations

BMI: body mass index
EI: energy intake
E%: energy percentage
FFQ: food frequency questionnaires
IQR: interquartile range
kJ: kilojoule
PAL: physical activity level
ρ: Spearman rank correlation
ρc: concordance correlation coefficient
